# Clinical Outcomes of Dual-Beam Particle Therapy in Head and Neck Adenoid Cystic Carcinoma

**DOI:** 10.3390/cancers18050753

**Published:** 2026-02-26

**Authors:** Gertrud Schmich, Alwina Keil, Fatima Frosan Sheikhzadeh, Fabian Eberle, Daniel Habermehl, Thomas Held, Philipp Lishewski, Boris A. Stuck, Hilke Vorwerk, Klemens Zink, Sebastian Adeberg, Ahmed Gawish

**Affiliations:** 1 Department of Radiotherapy and Radiation Oncology, Marburg University Hospital, 35037 Marburg, Germany; stalmang@staff.uni-marburg.de (G.S.); klemens.zink@lse.thm.de (K.Z.);; 2 Department of Radiotherapy and Radiation Oncology, Philips University, 35037 Marburg, Germany; 3 Department of Radiotherapy and Radiation Oncology, Marburg Ion-Beam Therapy Center (MIT), Marburg University Hospital, 35043 Marburg, Germany; 4 University Cancer Center (UCT) Frankfurt-Marburg, 35032 Marburg, Germany; 5 Mildred Scheel Early Career Center (MSNZ) for Cancer Research Marburg-Frankfurt, University Hospital Marburg, 35037 Marburg, Germany; 6 Department of Radiotherapy and Radiation Oncology, Giessen University Hospital, 35392 Giessen, Germany; 7 Department of Radiation Oncology, Heidelberg University Hospital, 69120 Heidelberg, Germany; 8 National Center for Tumor Diseases (NCT), NCT Heidelberg, A Partnership Between DKFZ and University Medical Center Heidelberg, 69120 Heidelberg, Germany; 9 Department of Otorhinolaryngology, Head and Neck Surgery, University Hospital Marburg, Philipps-Universität Marburg, 35043 Marburg, Germany; 10 LOEWE Research Cluster for Advanced Medical Physics in Imaging and Therapy (ADMIT), TH Mittel Hessen University of Applied Sciences, 35390 Giessen, Germany

**Keywords:** carbon ion, adenoid cystic carcinoma, particle therapy

## Abstract

**Background and Purpose**: This study retrospectively evaluates the outcomes of head and neck adenoid cystic carcinomas (ACCs) treated with particle therapy, including carbon ion radiotherapy (CIRT) alone or combined with photon therapy, at a single institution. **Methods and Materials**: Patients with ACC who underwent CIRT alone or a combination of CIRT and photon therapy at the Marburg Ion Therapy Center between February 2017 and December 2023 were included. Radiation therapy was administered postoperatively in surgically resectable patients and as definitive treatment in unresectable patients. Newly diagnosed patients received CIRT as a boost in combination with photon intensity-modulated radiation therapy (IMRT), while those with recurrent disease received CIRT alone. Prognostic factors were analyzed using Kaplan–Meier analysis and proportional hazards regression for multiple regression. Late toxicities (grade 3 or higher) were recorded according to the National Cancer Institute Common Terminology Criteria for Adverse Events, version 4. **Results**: A total of 73 patients were included, with a median age of 57 years (range: 16–86 years) and a median follow-up of 20 months (range: 3–70 months). The cohort included 28 males (38%) and 45 females (62%). The median CIRT dose was 24 Gy (relative biological effectiveness (RBE)) (range: 15–60 Gy) in a median of 8 fractions (range: 5–20), and the median photon dose was 50 Gy (range: 45–54 Gy) in 25 fractions (range: 15–30). Locoregional recurrence-free survival rates at 1 and 3 years were 89.6% and 75.4%, respectively, while distant metastasis-free survival rates were 82.1% and 61.4%, respectively. LC was significantly influenced by T stage, with patients with T4 tumors showing worse outcomes. Treatment was generally well tolerated, with acute side effects including mucositis and skin erythema. Severe chronic toxicities were rare, with only 1% of patients experiencing grade 3 dysphagia and grade 3 xerostomia. **Conclusions**: CIRT, particularly when combined with photon therapy, demonstrates favorable local control and promising efficacy in head and neck ACC, though distant metastasis remains the primary pattern of failure. Tumor stage is a significant negative prognostic factor for local control and overall survival.

## Simple Summary

Adenoid cystic carcinoma is a rare type of cancer characterized by locally aggressive growth and a high risk of distant metastases. Achieving durable local control is therefore a key goal of treatment. Standard local treatment options include surgery and radiation therapy. In this retrospective study, we analyzed outcomes of dual-beam radiation therapy using photons and carbon ions delivered at the Marburg Ion Therapy Center. The aim was to evaluate the clinical benefit of this combined radiation approach for patients with adenoid cystic carcinoma. The results showed favorable local control rates after combined particle and photon therapy. These findings suggest that dual-beam radiation therapy is an effective and beneficial local treatment option for patients with adenoid cystic carcinoma.

## 1. Introduction

Adenoid cystic carcinomas (ACCs), characterized by an incidence rate of 4.5 cases per 100,000 individuals and constituting merely 1% of all head and neck cancer types, are distinctly classified as a rare form of tumor which has a slight female predominance and a mean age of 57 years at diagnosis [1,2,3,4]. In Germany, population-based cancer registry analyses from large federal states show that ACC represents a minor histological subtype among major salivary gland carcinomas, with a stable incidence over time [5]. Although the salivary glands are the predominant site of onset, ACC can also manifest in various other locations within the head and neck region, including the external auditory canal, lacrimal glands, tongue, nasopharynx, and hard palate. Additionally, ACC can affect glands in the trachea, skin, breast, and reproductive tract [2,3,6]. ACC clinically presents as a slow-growing yet aggressive locoregional tumor with a tendency for perineural and lymphatic invasion. In the presence of distant metastasis, the lung and liver are the most frequently observed sites of involvement [7,8,9,10]. To date, the primary treatment for ACC remains complete surgical resection, with or without neck dissection; however, adjuvant radiotherapy has emerged as a crucial component of the therapeutic strategy. In specific cases, such as inoperability or palliative situations, primary radiotherapy or chemotherapy may serve as an alternative treatment option, though they are not considered first-line therapies [11,12,13,14].

**Figure 1:**
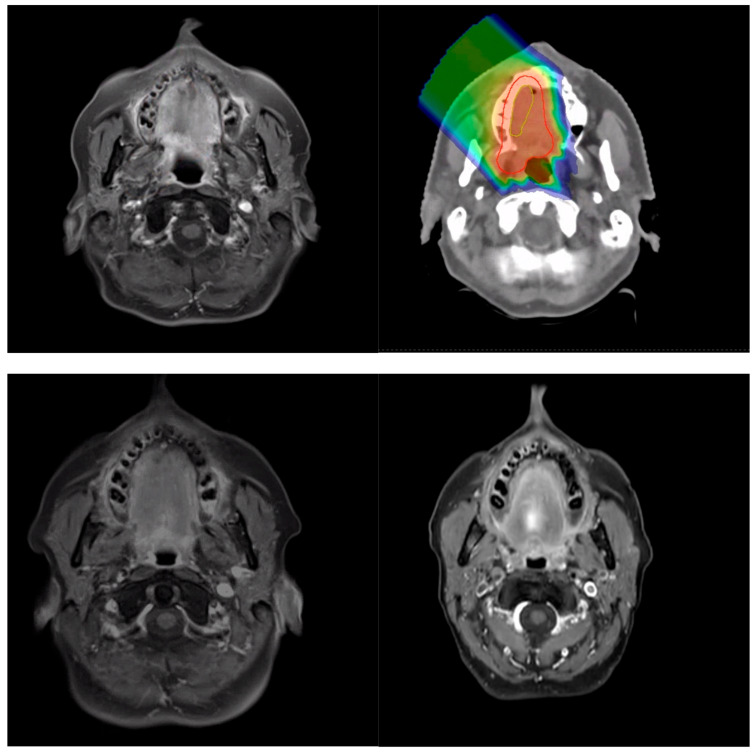
Radiologic tumor response following treatment. Tumor size changes at 12, and 24 months are shown relative to baseline imaging. Representative images from a single patient include the pre-treatment MRI and the corresponding radiotherapy plan, demonstrating target volume delineation and dose distribution.

In recent years, various modalities of radiotherapy have been employed in the treatment of ACC. Historically, photon radiation therapy was used; however, local control was limited, suggesting a rather radiation-resistant tumor type [15]. In addition to photon therapy, particle radiation therapy with fast neutrons, protons and carbon ions has been used. In previous studies, particle therapy with protons and carbon ions showed better local control compared to neutrons [15]. This study focuses particularly on the combination of photon therapy (PT) and CIRT due to their distinct physical properties and therapeutic potential. PT offers a cost-effective and widely implemented approach to radiotherapy. In contrast, the combination of photon beam therapy and CIRT necessitates specialized facilities and expertise, making it more expensive; however, it delivers a significantly more precise dose distribution, exemplarily visualized in Figure 1, primarily due to its physical properties, such as the Bragg peak, and is biologically more effective than photon radiation therapy due to its higher destructive power [16,17,18,19].

At the University of Marburg and Marburg Ion Beam Therapy Center, we are privileged to provide our patients with a variety of therapy strategies, including both PT and CIRT. In this retrospective study, our objective is to enhance clinical understanding of ACC treatment by evaluating the outcomes of CIRT alone and in combination with PT.

## 2. Methods and Materials

**Figure 2:**
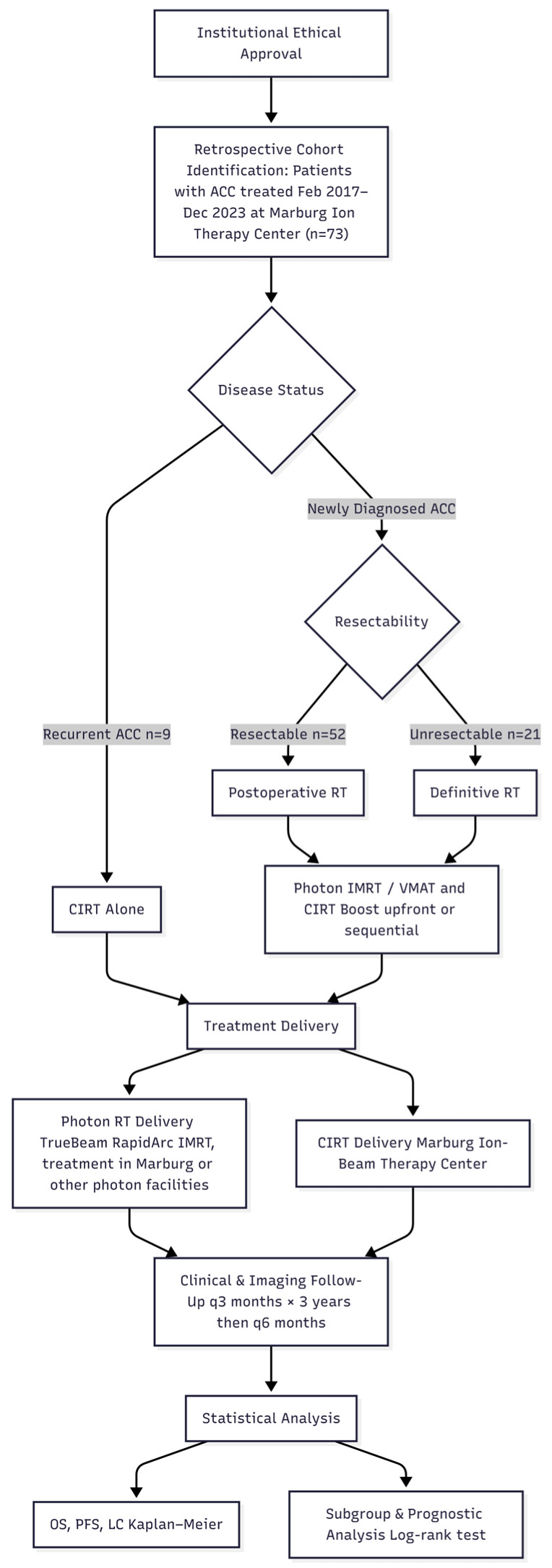
Flow diagram of patient selection, treatment allocation, and study methodology.

Figure 2 visualizes the study methodology. Following institutional ethical approval, we conducted a retrospective analysis of patients with ACC who underwent either CIRT alone or a combined modality treatment with C12 ions and photons at our center between February 2017 and December 2023.

Patients with newly diagnosed ACC received postoperative radiation therapy if the disease was surgically resectable, or definitive radiation therapy if unresectable. These patients were treated with a combined approach consisting of CIRT as a boost, delivered either upfront or sequentially with photon-based IMRT. In contrast, patients with recurrent disease were treated with CIRT alone. While CIRT was administered exclusively at our institution, photon-based IMRT or volumetric modulated arc therapy (VMAT) was performed either at our center or at a facility closer to the patient’s home.

### 2.1. Treatment Planning and Simulation

For both treatment delivery and planning computed tomography (CT), patients were immobilized using a customized thermoplastic head-and-shoulder mask to minimize movement and ensure precise treatment. A three-dimensional CT-based planning system was employed for treatment planning, with CT simulation performed using a slice thickness of 1–3 mm.

To reduce imaging artifacts in patients with metal implants, an iterative Metal Artifact Reduction (iMAR) algorithm was applied, resulting in improved image quality and more accurate anatomical delineation. Additionally, magnetic resonance imaging (MRI) was performed due to its superior soft tissue contrast, and the MRI datasets were rigidly registered with the planning CT to enhance target volume definition.

Particle beam dose was expressed as photon-equivalent dose in Gray (Gy), taking into account the relative biological effectiveness (RBE). For carbon ions, RBE values ranged from 2 to 3.7, depending on the location within the Bragg peak.

Treatment planning for CIRT was conducted using the Siemens Syngo RT VC13C_01030 planning software, while VMAT plans were generated using the Varian Eclipse version 15.6 planning system.

### 2.2. Target Volume Definition

Target volume definition for carbon ion irradiation was performed according to COSMIC protocol for the early patients; treatments after 12/2019 were planned according to ACCO target volume protocol [20,21].

The gross tumor volume (GTV-B) was identified as the contrast-enhancing primary tumor visible on T1-weighted contrast-enhanced MRI. For cases with nodal involvement, an additional gross tumor volume (GTV-SIB) containing the involved nodes was delineated. For each gross tumor volume, clinical target volumes (CTVs) were outlined. The clinical target volume for the CIRT boost (CTV-B) was created by expanding the GTV-B by 5 mm, respecting anatomical boundaries. The photon-based clinical target volume (CTV-photons) encompassed a broader area, including CTV-B, potential pathways of tumor spread, and, in advanced cases or those with nodal involvement, elective lymph node regions. For the simultaneous integrated boost (SIB), the clinical target volume (CTV-SIB) was defined as a 5 to 7 mm expansion around GTV-SIB.

Finally, the planning target volume (PTV) was established by adding a 3 mm margin around each respective CTV.

For patients undergoing reirradiation, the CTV was generated by expanding the GTV by 5–7 mm while respecting anatomical boundaries. An additional 3 mm margin was then applied to create the PTV.

### 2.3. Treatment

CIRT was delivered at the Marburg Ion Beam Therapy Center using active raster scanning with carbon ion beams and 2 to 4 non-coplanar beam angles. Patient positioning was verified with daily orthogonal X-ray image guidance, and weekly CT-based recalculations were performed to ensure treatment accuracy.

Photon radiotherapy was administered at the Department of Radiation Oncology, Marburg University Hospital, using a Varian TrueBeam linear accelerator equipped with a motorized multileaf collimator (MLC) with a 0.5 cm leaf width. Photon treatments were delivered using RapidArc IMRT technique, with daily image guidance via cone-beam CT (CBCT) in the treatment position.

**Figure 3:**
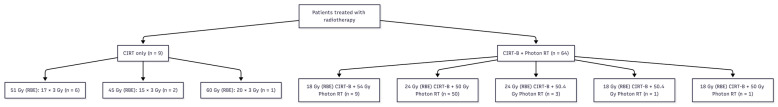
Treatment characteristics.

The prescribed dose was normalized to the median of the prescribed dose, and PTVs were covered by 95–107% of the isodose. Patients treated with CIRT alone received a total dose of 45–60 Gy (RBE) in 15–20 fractions. In patients receiving a carbon ion boost (CIRT-B), 18–24 Gy (RBE) was delivered in 6–8 fractions, followed by 50–54 Gy photon VMAT to the PTV-photons, delivered in daily fractions of 1.8 or 2.0 Gy (see Figure 3). All treatments were administered five times per week.

### 2.4. Pharmacological Treatment

Pharmacological therapy in this study cohort was heterogeneous. Nine patients (11%) received systemic treatment sequentially after radiation therapy at recurrence, consisting of either chemotherapy or immunotherapy; no systemic therapy was administered concurrently with radiation therapy. Among these, one patient received cisplatin and imatinib, three patients were treated with axitinib, and one patient each received doxorubicin and cyclophosphamide; nivolumab; pembrolizumab; lenvatinib and paclitaxel; or a combination of cisplatin, doxorubicin, and cyclophosphamide.

**Follow-up**

Follow-up assessments were conducted every three months during the first three years after completion of therapy and every six months thereafter. Routine follow-up included a comprehensive physical examination, endoscopic evaluation, and diagnostic imaging with CT and/or MRI. At each follow-up visit, a physical examination and endoscopic evaluation were performed. Cross-sectional imaging (MRI/CT of the primary site and/or CT of the thorax) was obtained in most follow-up assessments. The use and frequency of imaging depended on tumor visibility on imaging, clinical findings or symptoms, and patient consent. Acute and late treatment-related toxicities were assessed according to the Common Terminology Criteria for Adverse Events (CTCAE), version 4.0.

**Statistical Analysis**

Overall survival (OS), progression-free survival (PFS), and local control (LC) were estimated using the Kaplan–Meier method, which was selected to appropriately account for censored time-to-event data. OS was defined as the time from the start of radiotherapy to death from any cause or last follow-up. PFS was defined as the time from treatment initiation to disease progression or death, whichever occurred first. LC was defined as the time from treatment initiation to local tumor recurrence.

Comparative survival analyses were performed between two clinically relevant subgroups—patients with and without prior irradiation to the same anatomical region—using the log-rank test, which was chosen to assess differences in survival distributions between groups. In addition, univariate analyses of potential prognostic factors, including age, sex, tumor stage, and resection status, were conducted using the log-rank test to evaluate their association with OS, PFS, and LC.

All statistical tests were two-sided, and a *p*-value < 0.05 was considered statistically significant. Statistical analyses were performed using SPSS software (version 23.0; IBM Corp., Armonk, NY, USA), R version 4.3.2, and RStudio version 2024.04.2 +764 [22,23].

## 3. Results

### 3.1. Patient Characteristics

As shown in Table 1, 73 patients were included in this study, comprising 28 males (38%) and 45 females (62%), with a median age of 57 years (range: 16–86 years). The median follow-up duration was 20 months (range: 3–70 months). During this period, eight patients (11%) died. Tumor distribution across primary locations was as follows: oral cavity (33%), parotid gland (23%), paranasal sinus (16%), ear (7%), nasopharynx (5%), and nose (4%). Tumor staging revealed that 49.3% of patients had T4 tumors, 24.7% had T3 tumors, 9.6% had T2 tumors, and 8.2% had T1 tumors at initial diagnosis.

### 3.2. Treatment Characteristics

Of the total cohort, 21 patients (29%) underwent definitive radiation therapy, 8 patients (11%) received surgical tumor debulking followed by radiation therapy, and 23 patients (32%) had R1 resections prior to radiotherapy. Complete tumor resection was achieved before radiation in 21 patients (29%). The median CIRT dose was 24 RBE (range: 15–60 Gy), delivered in a median of 8 fractions (range: 5–20). For photon-based radiotherapy, the median dose was 50 Gy (range: 45–54 Gy), administered in a median of 25 fractions (range: 15–30).

Six patients (8.2%) received prior irradiation therapy in the same region; for these, a CIRT dose of 51 Gy RBE was delivered in 17 fractions without photon-based radiation therapy.

### 3.3. Survival and Disease Progression

**Figure 4:**
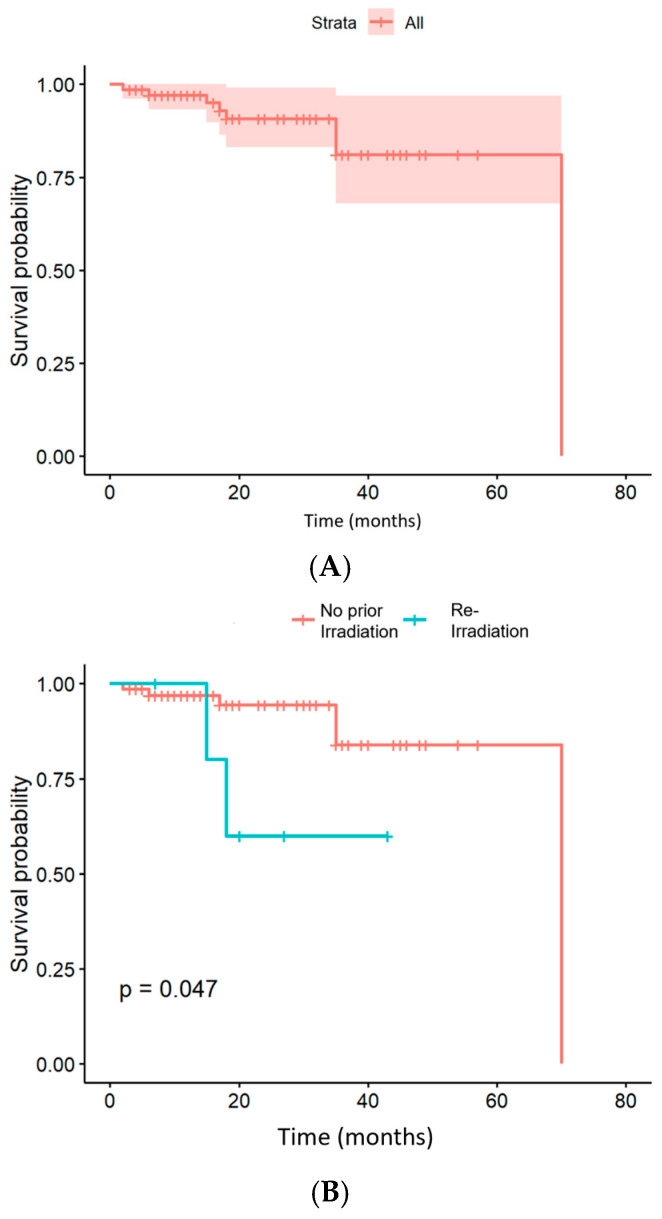
Kaplan–Meier estimation of OS after radiotherapy of 73 patients with ACC irradiated with CIRT. (**A**) OS independent of prior irradiation. (**B**) OS depending on reirradiation status.

At the conclusion of the follow-up period, 65 patients were alive. As shown in Figure 4, one-year OS rate was 97.1% (95% CI: 93.2–100%), two-year OS rate was 90.7% (95% CI: 83.1–99.0%), and three-year OS rate was 81.2% (95% CI: 68.0–96.9%). PFS had a median of 17 months (range: 2–57 months), with one-year, two-year, and three-year rates of 74.9%, 61.3%, and 56.6%, respectively (Supplementary Information, Figure S1). The median estimated LC duration following radiation therapy was 17 months (range: 2–57 months) (Supplementary Information, Figure S2). Tumor progression within the radiation field was observed in 11 patients, with a median time to progression of 14 months (range: 3–53 months). LC rates were 89.8% at one year and 75.8% at two years. Distant control rates were 82.3% at one year, 75.8% at two years and 63.2% at three years.

Figure S3 in the Supplementary Information shows the rate of distant metastasis after radiation treatment. At diagnosis, nine patients (12%) presented with distant metastases, located in the lungs (five patients, 7%), bones (two patients, 3%), and liver (two patients, 3%). Following treatment, 16 patients (22%) developed out-of-field metastases, including lung metastases in 11 patients (15%), bone metastases in 5 patients (7%), and liver metastasis in 5 patients (7%). Therefore, in total 16 patients (53% of all metastases) had lung metastasis, 7 patients (23% of all metastases) bone metastasis, and 7 patients (23% of all metastases) liver metastasis during the study period. The distant metastases developed after a median time of 12 months (range 2–67 months).

### 3.4. Treatment After Disease Progression

Local treatments after disease progression included radiation therapy for nine patients (12%) and surgery for six patients (8%). Radiation therapy in this context involved CIRT, with delivered doses including 51 Gy RBE (3 Gy RBE per fraction) for four patients, 48 Gy RBE (3 Gy RBE per fraction) for one patient, 60 Gy RBE (3 Gy RBE per fraction) for one patient, and 30 Gy RBE (3 Gy RBE per fraction) for one patient. These treatments were initiated at a median of 22 months after initial radiation therapy (range: 4–54 months).

### 3.5. Prognostic Factors for Survival and Local Control

Patients receiving primary radiotherapy (n = 67) demonstrated superior OS compared to those undergoing reirradiation (n = 6). The one-year and two-year OS rates were 96.8% and 94.5% for primary irradiation and 80.0% and 60.0% for reirradiation (*p* = 0.047). One-year and two-year PFS rates were 77.3% and 64.4% for primary radiation therapy and 50.0% and 33.0% for reirradiation (*p* = 0.045). LC was significantly different as well: the one-year and two-year LC rates were 92.2% and 79.0%, respectively, for primary radiation therapy and 62.5% and 41.7% for reirradiation (*p* = 0.059).

Another prognostic factor for PFS was the T status of the tumor: as shown in Figure S4, patients with T1-T3 tumor status had better PFS than those with T4 tumors (one-year and two-year PFS 84.1% vs. 64.6% and 79.4% vs. 41.5%, *p* = 0.0029). Furthermore, LC was correlated with T status: one- and two-year LC was 96.7% vs. 81.9% and 87.7% vs. 62.2%, respectively (*p* = 0.01).

Resection status, however, did not significantly impact OS in this cohort, as shown in Figure S5 (*p* = 0.11).

### 3.6. Treatment Safety and Tolerance

Table 2 shows that treatment was generally well-tolerated, with mucositis and skin erythema being the most commonly observed acute side effects. A notable eight patients (11%) experienced weight loss exceeding 5% of their initial body weight during the treatment course. Nutritional support was required in 26 patients (36%), with 18 patients (25%) receiving oral nutritional supplements and 8 patients (11%) requiring parenteral nutrition to manage treatment-related challenges.

Mild chronic side effects according to CTCAE v.4 were observed in several patients. Dysphagia was reported in 8 patients (11%), fatigue in 4 patients (5%), xerostomia in 22 patients (30%), and lymphedema in 8 patients (11%). Additionally, 17 patients (23%) experienced dysgeusia, and 1 patient (1%) reported anosmia.

Severe chronic side effects were rare, with only one patient (1%) experiencing grade 3 dysphagia and another patient (1%) reporting grade 3 xerostomia following radiation therapy. These findings suggest that while mild chronic side effects were relatively frequent, severe side effects occurred infrequently.

## 4. Discussion

Although high-LET radiation is more resource-intensive, recent studies suggest that the combination of IMRT with a carbon ion boost may be cost-effective in selected patient populations. Jensen and Debus conducted a cost-effectiveness analysis comparing IMRT plus CIRT to IMRT alone, demonstrating favorable economic metrics when long-term outcomes and toxicity reduction were considered [24]. While economic analyses remain limited, such data are essential to inform future reimbursement and policy decisions, particularly as access to CIRT expands. The results of our study, combined with insights from recent literature, support the significance of CIRT in the treatment of ACC of the head and neck—a rare and clinically challenging malignancy characterized by slow growth, perineural invasion, and a high risk of distant metastases.

Our cohort demonstrated encouraging LC rates, with one-year and two-year LC of 89.8% and 75.8%, respectively. These findings align with previously reported outcomes in both single- and multicenter trials. Akbaba et al. reported three- and five-year LC rates of 85% and 76%, respectively, in patients with sinonasal ACC treated with IMRT and an active raster-scanned CIRT boost [25]. Similarly, the Japanese J-CROS 1402HN multicenter study, which specifically analyzed CIRT in head and neck ACC, found LC rates above 80% at one year, supporting the efficacy of this modality [26]. Jensen et al. also demonstrated that the combination of IMRT and CIRT significantly improved locoregional control (LRC) and OS in advanced ACC patients, further corroborating our findings [27]. In this context, the term LRC refers to freedom from recurrence both at the primary site (local) and in the regional lymph nodes (regional) after treatment. In contrast, LC refers only to control of the disease at the primary tumor site itself (without considering regional lymph node involvement).

In terms of OS, our study reported one- and three-year OS rates of 97.1% and 81.2%, respectively, which are comparable to previously published reports that documented survival rates of approximately 80–85% over similar time intervals (Table 3, [15,25,26,27,28,29,30]). These outcomes underline the capacity of CIRT—either alone or in combination with IMRT—to achieve durable disease control and potentially prolong survival, particularly in patients with locally advanced or unresectable tumors.

Despite these favorable locoregional results, distant metastases continue to represent the predominant mode of treatment failure. In our cohort, 22% of patients developed new distant metastases following therapy, with the lungs being the most common site, followed by the liver and bones. This emphasizes the importance of thoracic CT for follow-up. These patterns are consistent with those reported by Akbaba et al., Sulaiman et al., and Jensen et al., who also observed high rates of distant progression despite excellent LC [25,26]. The persistence of distant failure highlights the systemic nature of ACC and underscores the need for integrated treatment strategies. CIRT effectively addresses locoregional disease, which is the leading cause of symptoms, while systemic metastases often follow an indolent course, making initial locoregional treatment essential. However, achieving locoregional control does not mitigate the risk of hematogenous dissemination, which remains a significant threat to long-term survival.

Consequently, the potential benefit of combining CIRT with systemic therapies—such as chemotherapy, targeted agents (e.g., tyrosine kinase inhibitors), or immune checkpoint inhibitors—warrants further investigation. Recent studies have focused on targeted therapies and immuno-oncologic approaches because of the limited efficacy of chemotherapy and immune checkpoint inhibitors. However, to date, no standardized treatment has been established for this rare, slow-growing, yet ultimately fatal disease [32]. In our study, only 11% of patients received systemic therapy after the diagnosis of recurrence, and outcomes in this subgroup were not analyzed separately due to the small sample size and treatment heterogeneity. Nonetheless, integrating CIRT with systemic therapies represents a promising strategy to mitigate the burden of distant relapse, particularly in high-risk patients.

Our analysis also identified key prognostic factors. Notably, tumor stage significantly influenced both LC and PFS. Patients with T1–T3 tumors demonstrated superior outcomes compared to those with T4 tumors (PFS at 1 and 2 years: 84.1% vs. 64.6% and 79.4% vs. 41.5%, *p* = 0.0029), consistent with the literature [25,26,33,34]. The same trend was observed for LC, highlighting the adverse prognostic impact of advanced primary tumor extent. These findings reinforce the importance of early diagnosis and timely initiation of radiotherapy.

Interestingly, resection status did not significantly impact OS in our cohort (*p* = 0.11). This observation aligns with the study by Lloyd et al., which analyzed 2286 cases of surgically treated head and neck ACC and found that complete resection alone did not significantly improve overall or cause-specific survival [35]. Instead, factors such as tumor extent (distant metastasis, lymph node involvement, higher T classification) and older age were more strongly associated with poor outcomes. These data support the role of adjuvant radiotherapy—even in R0-resected patients—as an integral component of ACC treatment, particularly when delivered using high-LET radiation.

From a safety perspective, our data confirm the favorable toxicity profile of CIRT. Acute toxicities, including mucositis and dermatitis, were common but manageable. Severe late toxicities (CTCAE grade ≥3) were rare, with only 1% of patients experiencing grade 3 dysphagia or xerostomia. The majority of chronic side effects were mild to moderate, including fatigue, lymphedema, and xerostomia. These findings are in line with previous studies demonstrating low rates of severe toxicity with raster-scanned carbon ion therapy [33,34,36]. The high conformality of dose distribution and steep dose gradients afforded by CIRT likely contribute to the reduction in toxicity, particularly in anatomically complex regions such as the skull base, orbit, or nasopharynx.

Long-term experiences from centers such as Heidelberg [34] have provided additional reassurance regarding the safety and durability of CIRT in ACC patients, with consistently high LC rates and low toxicity over more than 15 years of follow-up [34]. The biological rationale for using carbon ions—high linear energy transfer (LET), increased RBE, and lower oxygen enhancement ratio (OER)—further supports their application in radioresistant tumors like ACC [37]. The combination of IMRT and CIRT represents a rational and technically feasible strategy to maximize LC while minimizing adverse effects.

Nevertheless, this study has several limitations. First, the retrospective design is inherently prone to selection and reporting biases. Second, the relatively short median follow-up (20 months) limits conclusions about long-term survival and late toxicity. Third, the heterogeneity of the cohort—including variability in surgical status, prior irradiation, and systemic therapy—may confound the interpretation of clinical outcomes. Fourth, although the number of patients treated with CIRT is relatively high for this rare tumor, it may nonetheless be inadequate to statistically discriminate the impact of factors such as resection margin status. Fifth, due to the limited number of centers capable of delivering CIRT, this study was conducted at a single institution, which may introduce center-specific bias and limit the generalizability of the results. Despite these limitations, the present study contributes important real-world data on the feasibility, safety, and effectiveness of CIRT in a contemporary cohort of head and neck ACC patients.

Looking ahead, prospective trials are essential to refine patient selection, treatment protocols, and follow-up strategies. Ongoing trials such as ACCO (evaluating carbon ion monotherapy) and ETOILE (comparing carbon ion therapy to conventional radiotherapy) will provide critical evidence to guide future clinical practice [20,21,38]. Moreover, translational research into molecular predictors of metastasis and response to CIRT could facilitate personalized treatment strategies. The integration of CIRT with systemic agents—including novel immunotherapies or targeted treatments—may ultimately improve outcomes for patients at high risk of systemic progression.

## 5. Conclusions

In this retrospective analysis, CIRT administered alone or in combination with pho-ton-based IMRT was associated with favorable LC and acceptable toxicity in patients with head and neck adenoid cystic carcinoma, including those with locally advanced, recurrent, or unresectable disease. The dual-beam approach combining carbon ions with photon radiotherapy may represent a clinically pragmatic strategy that integrates the biological properties of carbon ions with established photon-based techniques. However, given the retrospective design and the absence of comparative and health-economic analyses, no definitive conclusions regarding relative efficacy or cost-effectiveness can be drawn.

Tumor stage and prior irradiation were identified as significant prognostic factors, supporting individualized, risk-adapted treatment strategies. Despite satisfactory LC, dis-tant metastases remain a predominant pattern of failure. Prospective studies are war-ranted to confirm these findings and to further evaluate long-term outcomes, optimal therapy sequencing, comparative effectiveness, and economic aspects of carbon ion-based treatment approaches.

## Figures and Tables

**Table 1:** Patient characteristics.

Category	Details
**Patient Characteristics**	
Total Patients	73
Gender Distribution	28 males (38%), 45 females (62%)
Median Age (Range)	57 years (16–86 years)
**Median Follow-up Duration (Range)**	**20 months (3–70 months)**
**Deaths During Follow-up**	**8 patients (11%)**
**Tumor Locations**	
Oral Cavity	33%
Parotid Gland	23%
Paranasal Sinus	16%
Ear	7%
Nasopharynx	5%
Nose	4%
**Treatment Characteristics**	
Definitive Radiation Therapy	21 patients (29%)
Surgical Tumor Debulking + Radiation	8 patients (11%)
R1 Resection + Radiation	23 patients (32%)
Complete Resection + Radiation	21 patients (29%)
**Tumor Stages (T1–T4)**	
T1	8.20%
T2	9.60%
T3	24.70%
T4	49.30%
Median CIRT Dose (Range)	24 RBE (15–60 Gy), Median Fractions: 8 (5–20 fractions)
Median Photon Therapy (Range)	50 Gy (45–54 Gy), Median Fractions: 25 (15–30 fractions)
**Local Control and Survival**	
Alive at Follow-up	65 patients
Median Local Control	17 months (2–57 months)
Progression within Radiation Field	11 patients, Median Time: 14 months (3–53 months)
Progression out of Field/Distant Metastases	19 patients (26%), Median Time: 16 months (1–49 months)
Locoregional Recurrence-Free Survival	1 Year: 89.6%, 3 Years: 75.4%
Distant Metastasis-Free Survival	1 Year: 82.1%, 3 Years: 61.4%
**Distant Metastasis/Progression**	
Pre-treatment Distant Metastases	9 patients (12%)
- Lung	5 patients (7%)
- Bone	2 patients (3%)
- Liver	2 patients (3%)
**Post-treatment Out-of-Field Metastases**	**16 patients (22%)**
- Lung	11 patients (15%)
- Bone	5 patients (7%)
- Liver	5 patients (7%)
**Local Treatment After Progression**	**Radiation Therapy: 9 patients (12%), Surgery: 6 patients (8%)**
**Radiation Doses for Progression**	**4 patients: 51 Gy RBE (3 Gy RBE/fraction), 1 patient: 48 Gy RBE (3 Gy RBE/fraction)**

**Table 2:** Absolute number and relative number of acute and late adverse events after radiotherapy of 73 patients with ACC irradiated with CIRT-B combined with VMAT according to common-toxicity CTCAE V 4.0.

	Acute Side Effects				Chronic Side Effects		
CTCAE grade	I	II	I/II	III	I	II	III
Dermatitis [n/%]	27/36.99%	11/15.07%	38/52.06%	-	3/4.11%		
Mucositis [n/%]	14/19.18%	11/15.07%	25/34.25%	-	3/4.11%	2/2.7%	0
Dysphagia [n/%]			20/27.4%	8/10.96%	7/9.59%	2/2.74%	1/1.37%
Dysgeusia [n/%]			7/9.59%	-			
Dry mouth [n/%]			8/10.96%	-	14/19.18%	7/9.59%	1/1.37%
Fatigue [n/%]			2/2.74%	-	6/8.22%		

**Table 3:** Study characteristics of studies with ≥50 patients treated with CIRT alone, IMRT plus CIRT boost, or proton beam therapy [15,25,26,27,28,29,30,31].

Study Details	Patient Number/Tumor Burden	Surgery	Beam Type	OS	LC
Evaluation of the safety and efficacy of carbon ion radiotherapy for locally advanced adenoid cystic carcinoma of the tongue base. Koto et al. 2016 [27]	18 patients, T1–3: 6%, T4: 94%, N0 61%, N1: 33%; N2b: 6%	NA	CIRT 57.6 Gy(RBE)/16 Fx	3-year: 100%, 5-year: 72%	3-year: 100%, 5-year: 92%
Multicenter Study of Carbon-Ion Radiation Therapy for Adenoid Cystic Carcinoma of the Head and Neck: Subanalysis of the Japan Carbon-Ion Radiation Oncology Study Group (J-CROS) Study (1402 HN). Sulaiman et al. 2018 [26]	289 patients, T1–3: 29%, T4: 69%	yes: 19%, no: 81%	CIRT 64 Gy(RBE)/16 Fx	2-year: 94%, 5-year: 74%	2-year: 88%, 5-year: 68%
Prognostic factors of adenoid cystic carcinoma of the head and neck in carbon-ion radiotherapy: The impact of histological subtypes. Ikawa et al. 2017 [28]	100 patients, T1–3: 27%, T4: 61%, N0: 94%, N1: 5%, N2 1%, M0: 100%	yes: 15% no: 85%	CIRT 64 Gy RBE/16 Fx	5-year: 74.8%	5-year: 68.8%
Impact of Perineural Tumor Spread in Head and Neck Adenoid Cystic Carcinoma for Carbon-Ion Radiotherapy. Musha et al. 2025 [29]	74 patients, T1–T3: 31%, T4; 69%, N0 93%, N+ 7%, M0: 81%, M1: 19%	yes: 11% no: 89%	CIRT 57.6 Gy (RBE) (28%), 64 Gy (RBE) (72%)	3-year: 89.4%, 5-year: 79%	3-year: 90.5%, 5-year: 67.6%
Treatment Outcome of 227 Patients with Sinonasal Adenoid Cystic Carcinoma (ACC) after Intensity Modulated Radiotherapy and Active Raster-Scanning Carbon Ion Boost: A 10-Year Single-Center Experience. Cancers (Basel). Akbaba et al. 2019 [25]	227 patients, T1–3: 20.7%, T4: 79.3%, N0 87.7%, N1: 4.4%, N2: 7.9%, M0: 98.7%, M1: 1.3%	yes: 60% no: 40%	IMRT 48–56 Gy (1.8–2 Gy); CIRT boost 18–24 Gy (RBE) (3 Gy (RBE))	3-year: 64% (primary RT), 79% (postoperative)	3-year: 79% (primary RT), 82% (postoperative)
Combined intensity-modulated radiotherapy plus raster-scanned carbon ion boost for advanced adenoid cystic carcinoma of the head and neck results in superior locoregional control and overall survival. Jensen et al. 2019 [24]	58 patients, T1–3:10%, T4 90%, N+: 7%, M1: 7%; Relapse therapy 31%, primary treatment 69%	yes: 69% no: 31%	IMRT+CIRT boost; Prescription 72 Gy (RBE)	3-year: 89.6%, 5-year: 76.5%	3-year: 83.7%, 5-year: 42.2%
Long-Term Outcomes Following Definitive or Adjuvant Proton Radiotherapy for Adenoid Cystic Carcinoma. Augustin et al. 2024 [30]	56 patients, T1–3: 37.5%, T4: 62.5%	yes: 84% no: 16%	Proton beam therapy 72.6 Gy (RBE), accelerated hyperfractionated 1.2 Gy (RBE) bid	5-year: 78%	5-year LRC 88%
Proton Therapy for Head/Neck Adenoid Cystic Carcinoma. Youssef et al. 2024 [31]	128 patients, Adjuvant: T1–3: 63%, T4: 37%, N0: 86%, N+: 19%, M14% Definitive T1–3: 13%, T4: 87%, N0: 84%, N+: 16%, M1: 39%	Yes: 28% no: 72%	Adjuvant 66 Gy (RBE), Definitive 70 Gy (RBE)	Adjuvant: 2-year: 92% 4-year: 90% Definitive: 2-year: 87% 4-year: 54%	Adjuvant: 2-year: 96% 4-year: 91% Definitive: 2-year: 90% 4-year: 82%

## Data Availability

The data presented in this study are available on request from the corresponding author.

## Supplementary Materials

The following supporting information can be downloaded at https://www.mdpi.com/article/10.3390/cancers18050753/s1. Figure S1. Kaplan–Meier estimation of progression-free survival after radiotherapy of 73 patients with ACC irradiated with CIRT combined with VMAT or IMRT. (A) PFS independent of reirradiation status. (B) PFS depending on reirradiation status. Figure S2. Kaplan–Meier estimation of local control after radiotherapy of 73 patients with ACC irradiated with CIRT combined with VMAT or IMRT. (A) LC independent of reirradiation status. (B) LC depending on reirradiation status. Figure S3. Kaplan–Meier estimation of distant metastasis after radiotherapy of 73 patients with ACC irradiated with CIRT combined with VMAT or IMRT. Figure S4. Kaplan–Meier estimation of local control depending on T4 status. Figure S5. Kaplan–Meier estimation of local control depending on resection status.

## Abbreviations

ACC	Adenoid cystic carcinoma
C12	Carbon ion
CIRT	Carbon ion radiotherapy
CBCT	Cone beam computed tomography
CI	Confidence interval
CTV	Clinical target volume
CTCAE	Common terminology criteria for adverse events
CTV-photon	Clinical target volume for photon IMRT
CTV-B	Clinical target volume for CIRT boost
CTV-SIB	Clinical target volume—simultaneous integrated boost
CT	Computed tomography
Gy	Gray
GTV	Gross tumor volume
GTV-LN	Gross tumor volume—pathological lymph nodes
GTV-SIB	Gross tumor volume—simultaneous integrated boost
GTV-B	Gross tumor volume—boost
GTV-A	Contrast enhanced primary tumor in MRI
iMAR	Iterative metal artifact reduction
LC	Local control
MRI	Magnetic resonance imaging
OS	Overall survival
PEG	Percutaneous endoscopic gastrostomy
PTV	Planning target volume
PFS	Progression-free survival
RBE	Relative biological effectiveness
VMAT	Volumetric modulated arc therapy
