# Supplementary material for: Clinical Outcomes of Dual-Beam Particle Therapy in Head and Neck Adenoid Cystic Carcinoma
Source: Cancers (Basel). 2026 Feb 26;18(5):753. doi: 10.3390/cancers18050753 (PMC12984363; doi:10.3390/cancers18050753)
Supplement: Supplementary file 1 [file cancers-18-00753-s001.zip › cancers-4129795-supplementary.pdf]

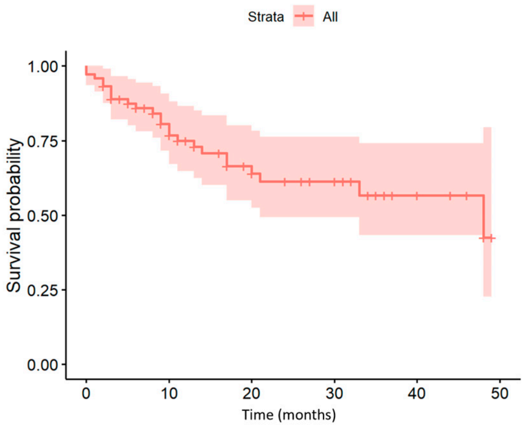

(**A**)

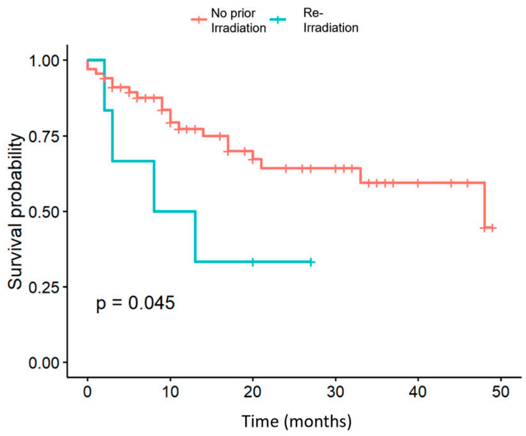

(**B**)

Figure S1. Kaplan-Meier estimation of progression free survival after radiotherapy of 73 patients with ACC irradiated with CIRT combined with VMAT or IMRT. (A) PFS independent of reirradiation status. (B) PFS depending on reirradiation status.

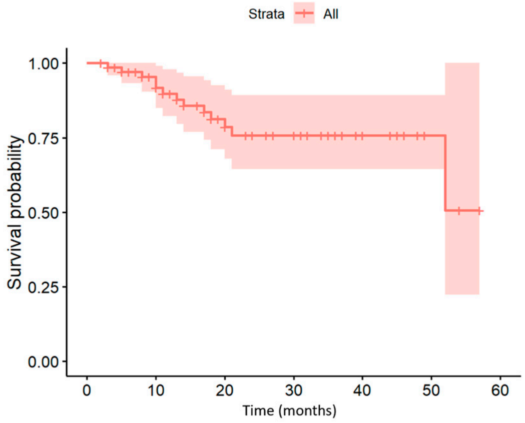

(a)

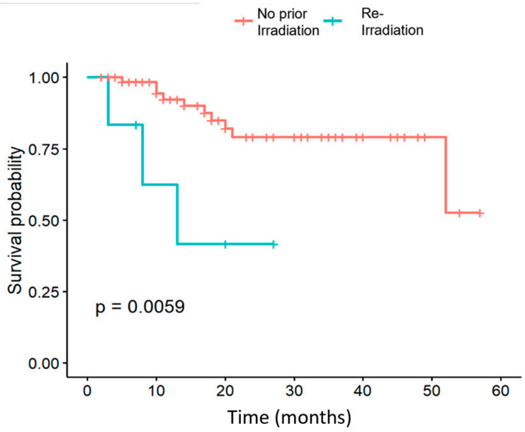

(b)

Figure S2. Kaplan-Meier estimation of local control after radiotherapy of 73 patients with ACC irradiated with CIRT combined with VMAT or IMRT. (A) LC independent of reirradiation status. (B) LC depending on reirradiation status.

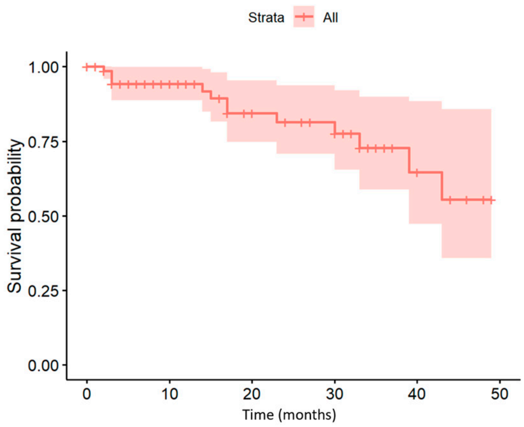

Figure S3. Kaplan-Meier estimation of distant metastasis after radiotherapy of 73 patients with ACC irradiated with CIRT combined with VMAT or IMRT.

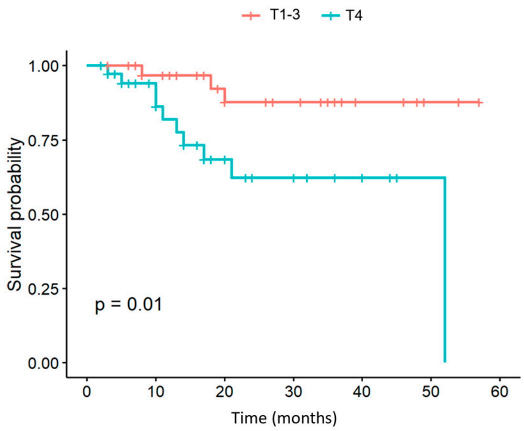

Figure S4. Kaplan Meier estimation of local control depending on T4 status.

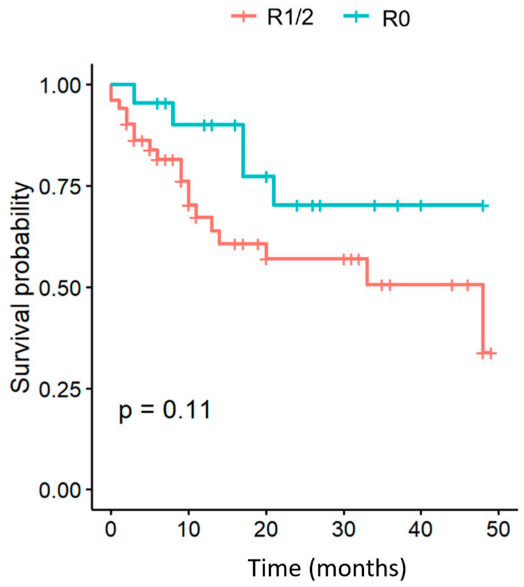

Figure S5. Kaplan Meier estimation of local control depending on resection status.
